# Rapid screening of IgG quality attributes – effects on Fc receptor binding

**DOI:** 10.1002/2211-5463.12283

**Published:** 2017-09-05

**Authors:** Karin P. M. Geuijen, Cindy Oppers‐Tiemissen, David F. Egging, Peter J. Simons, Louis Boon, Richard B. M. Schasfoort, Michel H. M. Eppink

**Affiliations:** ^1^ Downstream processing Synthon Biopharmaceuticals BV Nijmegen the Netherlands; ^2^ Bioprocess Engineering Wageningen University the Netherlands; ^3^ Preclinical department Synthon Biopharmaceuticals BV Nijmegen the Netherlands; ^4^ Bioceros BV Utrecht the Netherlands; ^5^ Medical Cell Biophysics group MIRA institute Faculty of Science and Technology University of Twente Enschede the Netherlands

**Keywords:** biolayer interferometry, Fcγ receptor, high‐throughput screening, in‐process control, neonatal Fc receptor, surface plasmon resonance imaging

## Abstract

The interactions of therapeutic antibodies with fragment crystallizable γ (Fcγ) receptors and neonatal Fc receptors (FcRn) are measured *in vitro* as indicators of antibody functional performance. Antibodies are anchored to immune cells through the Fc tail, and these interactions are important for the efficacy and safety of therapeutic antibodies. High‐throughput binding studies on each of the human Fcγ receptor classes (FcγRI, FcγRIIa, FcγRIIb, FcγRIIIa, and FcγRIIIb) as well as FcRn have been developed and performed with human IgG after stress‐induced modifications to identify potential impact *in vivo*. Interestingly, we found that asparagine deamidation (D‐N) reduced the binding of IgG to the low‐affinity Fcγ receptors (FcγRIIa, FcγRIIb, FcγRIIIa, and FcγRIIIb), while FcγRI and FcRn binding was not impacted. Deglycosylation completely inhibited binding to all Fcγ receptors, but showed no impact on binding to FcRn. On the other hand, afucosylation only impacted binding to FcγRIIIa and FcγRIIIb. Methionine oxidation at levels below 7%, multiple freeze/thaw cycles and short‐term thermal/shake stress did not influence binding to any of the Fc receptors. The presence of high molecular weight species, or aggregates, disturbed measurements in these binding assays; up to 5% of aggregates in IgG samples changed the binding and kinetics to each of the Fc receptors. Overall, the screening assays described in this manuscript prove that rapid and multiplexed binding assays may be a valuable tool for lead optimization, process development, in‐process controls, and biosimilarity assessment of IgGs during development and manufacturing of therapeutic IgGs.

**Abbreviations**
ADCC: antibody‐dependent cellular cytotoxicity
ADCP: antibody‐dependent cellular phagocytosis
BLI: biolayer interferometry
CDR: complementary‐determining region
CFM: continuous‐flow microspotter
CHO: chinese hamster ovary
CQA: critical quality attribute
DSS: disuccinimidyl suberate
EDC: 1‐ethyl‐3‐(3‐dimethylaminopropyl)carbodiimide hydrochloride
FcRn: neonatal Fc receptor
HC‐CDR: heavy chain complementary‐determining region
HC‐Fc: heavy chain Fc region
HEK: human embryo kidney
HMW: high molecular weight
HRP: horseradish peroxidase
IMAC: immobilized metal affinity chromatography
LC‐CDR: light chain complementary‐determining region
NHS: *N*‐hydroxysuccinimide
RU: resonance unit
SEC: size exclusion chromatography
SPR: surface plasmon resonance
TFA: trifluoroacetic acid

Therapeutic antibodies, like IgGs, are one of the largest classes of modern biopharmaceuticals, and the market for these products continues to grow year by year 1. Interactions of IgGs with effector cells through fragment crystallizable γ (Fcγ) receptors are often considered a mode of action of therapeutic antibodies 2, 3, 4. Fcγ receptors are cell surface receptors that can be found on innate immune effector cells such as natural killer cells and macrophages. A therapeutic IgG binds to a membrane‐bound antigen on target cells by its complementary‐determining regions (CDRs) in the variable domain, while the Fc region in the constant domain of that same IgG can bind to various Fcγ receptors on effector cells, which could lead to effector function, like antibody‐dependent cellular cytotoxicity (ADCC) or phagocytosis (ADCP). Therefore, binding of therapeutic antibodies to Fcγ receptors should be evaluated as part of the critical quality attribute (CQA) assessment 2.

Different Fcγ receptor subclasses are known to be present on human effector cells: the high‐affinity FcγRI (CD64) and the low‐affinity receptors FcγRIIa (CD32a), FcγRIIb (CD32b), FcγRIIIa (CD16a), and FcγRIIIb (CD16b) 5, 6. Within these five subclasses, different polymorphic variants exist, which, in some cases, influence binding of IgG to these receptors 7. Furthermore, the neonatal Fc receptor (FcRn) determines the half‐life of IgGs in the bloodstream. Binding of Fc receptor to IgG takes place in the endosome at acidic pH, and the IgG is then recycled back into plasma at neutral pH, thereby preventing lysosomal degradation. Recent studies have investigated the correlation between the *in vitro* binding of IgGs to FcRn and their corresponding serum half‐life 8, 9. Datta‐Mannan *et al*. 10 suggest that the *in vitro–in vivo* correlation of the FcRn binding cannot always directly be made, as IgG target binding may influence elimination of the IgG from the system as well. FcRn does not belong to the Fcγ receptor subclasses and binds to a different region in the IgG 11 than IgG regions recognized by Fcγ receptors. We will refer to Fc interactions as a general term, which includes both the Fcγ interactions and FcRn interactions.

Therapeutic IgGs are prone to many different post‐translational modifications during production and processing, which may have an impact on the Fc tail functionality. Monitoring the levels of modifications throughout the entire development, production, and marketing of IgGs is required from a regulatory perspective. Several modifications on IgGs are known to affect the binding to Fc receptors, such as aglycosylation 12, 13, 14, 15, 16, differential glycosylation (i.e., galactosylation 12, 14, 15, sialylation 12, and fucosylation 13, 16, 17, 18, 19), methionine oxidation (Ox) 20, 21, 22, 23, and aggregation 15, 23, 24, 25, 26, 27. We investigated the effects of these modifications, and additionally looked into effects of D‐N, heat/shake stress, and repeated freeze/thaw cycles (FT) on IgGs to Fc receptor binding. Stress studies were performed to accelerate modifications on an IgG1, and these were measured on all Fc receptors and quantified by HPLC, CE, or mass spectrometry as a reference method. Modifications that were introduced were kept at levels that are likely to be expected during actual in‐process measurements or shelf life studies, that is, generally not higher than 10% modification.

The aim of our study was to develop a screening assay that would rapidly measure IgG binding to the different Fcγ receptors and FcRn as part of CQA assessments during lead optimization studies and in‐process control. However, the biological differences in binding properties between Fc receptors prevented the development of a single screening sensor. Affinity ranges of FcRn and FcγRI (nm) compared to FcγRIIIa, FcγRIIIb, FcγRIIa, and FcγRIIb (μm) limited the analysis of IgGs in proper concentration ranges for each of the Fc receptor in a single measurement. On top of that, kinetics of IgG binding to FcRn follow a completely different profile (association at pH 6, dissociation at both pH 6 and pH 7.4) compared to the other Fcγ receptors (association and dissociation at pH 7.4) and this could not be combined into a single assay. Therefore, Fcγ receptor interactions of FcγRIIa, FcγRIIb, FcγRIIIa, and FcγRIIIb were simultaneously measured in a surface plasmon resonance (SPR) imaging setup, while a separate SPR method for FcγRI binding and a biolayer interferometry (BLI) method for FcRn binding were developed, all aimed at rapid measurements of IgG samples for high‐throughput screening purposes.

Two possible assay setups were considered: Fc receptor or IgG immobilization as ligand at the sensor surface. Preferably, the Fc receptors are used as ligand at the sensor surface, as this may best reflect the binding of Fc receptor to IgG *in vivo*, with Fc receptors present at cell surfaces. However, limited receptor stability of Fcγ receptors at the sensor surface (K. de Laat‐Arts & D. Egging, unpublished results) is most likely the reason why most literature about SPR‐based or BLI‐based Fcγ receptor binding studies is based on either capture approaches where fresh ligand is captured each cycle 12, 15, 16, 24, 25 or where IgG is immobilized at the sensor surface followed by Fcγ receptor injections 12, 28. We have developed a rapid multiplexed SPR sensor with the Fcγ receptors captured by biotin–streptavidin capture where ligand instability was mitigated. This method was qualified for proper performance, followed by analysis of stressed IgG samples to investigate the effects of IgG degradation on previously mentioned stress conditions on Fcγ receptor binding. The same stressed IgG samples were furthermore analyzed on the screening assays for FcγRI and FcRn. We found effects of deamidation on Fc receptor binding that, to the author's knowledge, have not been described previously in the literature.

## Materials and methods

### Recombinant proteins

The monoclonal antibody, a human IgG1, was produced and purified by Synthon Biopharmaceuticals BV. IgG1 samples with aberrant fucosylation profiles were a kind gift from Bioceros BV. The IgG samples from both sources have the same amino acid sequence and were produced in chinese hamster ovary (CHO) cells.

Human Fcγ receptors FcγRIIIa, FcγRIIIb, FcγRIIa, and FcγRIIb were produced in a human embryo kidney 293 (HEK293) expression system at Synthon Biopharmaceuticals BV. Receptors were expressed with a C‐terminal His‐tag followed by immobilized metal affinity chromatography (IMAC) purification as previously described 29. Human FcRn (human FCGRT & B2M heterodimer) and human FcγRI with a C‐terminal AVI‐tag and C‐terminal His‐tag were purchased from Sino Biological.

### Preparation of stressed human IgG samples

IgG1 samples were exposed to accelerated Ox by mixing 200 μL of 25 mg·mL^−1^ IgG1 with 4 μL (0.1%), 10 μL (0.25%) or 20 μL (0.5%) 5% H_2_O_2_ (Sigma‐Aldrich) and kept at room temperature for 10 min. Then, 5 μL catalase (4 U; Sigma‐Aldrich) was added and kept at room temperature for 5 min.

Accelerated deamidation was induced on the IgG1 by keeping the protein in 50 mm sodium phosphate buffer pH 8 at 20 mg·mL^−1^ for 48, 72, or 96 h at 40 °C. Samples were neutralized to pH 7.2 after incubation. As a control, samples were placed at the same temperature and time in neutral pH [HEPES‐buffered saline (HBS) buffer pH 7.2].

Thermal/shake stress was performed on the IgG1 samples by placing them at 40 °C at 1000 r.p.m. in HBS buffer pH 7.2 for 1, 4, 24, 32, 48, or 72 h. Another thermal/shake stress was applied by placing the IgG1 samples at 70 °C or 75 °C for 15 min at 300 r.p.m.

Freeze/thaw stress was applied by placing 250 μL of IgG1 at 25 mg·mL^−1^ in HBS pH 7.2 buffer at −80 °C. Samples were thawed and frozen again from 1 up to 10 FT in total.

The IgG1 sample was deglycosylated by mixing 50 μL sample (25 mg·mL^−1^) with 130 μL 200 mm sodium phosphate buffer pH 6.8. Then, 20 μL PNGase F solution was added and the solution was incubated at 37 °C for 24 h.

### Characterization of stressed samples

The levels of methionine Ox and D‐N in the stressed samples were determined using a tryptic peptide mapping followed by separation on a reversed‐phase C18 column. 0.05% trifluoroacetic acid (TFA) in MQ and 0.05% TFA in 50 : 50 MQ/acetonitrile were used as mobile phases A and B, respectively, and a linear gradient from 20% B to 99% B was used. Either UV or MS detection was used for quantitation. Percentages of methionine Ox or D‐N were calculated relative to the corresponding unmodified peptide.

Aggregation levels were determined based on a size exclusion chromatography (SEC)‐HPLC separation. The deglycosylated sample was checked for complete removal of glycans using CE‐SDS under nonreducing conditions.

Antigen target binding was verified on a Biacore T200 instrument (GE Life Sciences, Eindhoven, the Netherlands). Recombinant human antigen (R&D systems) was immobilized on a CM5 chip (GE life sciences) at 2.5 μg·mL^−1^ in sodium acetate pH 4.0. MabSelect SuRe (GE Life Sciences) was immobilized on the same sensor at 40 μg·mL^−1^ in sodium acetate pH 4.5 for total IgG1 determination. Contact times of 1200 and 360 s were applied, respectively, and immobilization was performed at 25 °C. IgG1 binding to antigen target and MabSelect SuRe were determined at 37 °C with an association time of 42 s and dissociation time of 30 s and a flow rate of 10 μL·min^−1^. Regeneration was performed with 10 mm glycine/HCl pH 1.5 with a contact time of 30 s and flow rate of 30 μL·min^−1^. Antigen target binding was expressed as binding relative to a reference sample which was set at 100% binding. Data of the MabSelect SuRe surface were only included to verify appropriate IgG concentrations in case of reduced antigen target binding. All sensorgrams were referenced and zeroed during data analysis.

Antigen target binding of the aberrant fucosylated samples was determined in an ELISA format. The antigen was coated in flat‐bottomed half‐area 96‐well clear polystyrene plates at 0.75 μg·mL^−1^ in PBS pH 7.2. After blocking with 1% w/v BSA, serially diluted IgG samples and references were added followed by a detection step with 1 : 5000‐diluted horseradish peroxidase (HRP)‐labeled goat anti‐human IgG Fcγ‐specific antibodies. Optical densities were read at 450 nm after development with a ready‐to‐use tetramethylbenzidine solution according to the manufacturer's instructions (Thermo Fisher Scientific Inc) using an ELISA reader (Bio‐Rad Laboratories, Hercules, CA, USA). All binding reactions were performed at room temperature in the presence of 1% w/v BSA and 0.05% v/v Tween‐20 detergent.

Fucosylation levels of aberrantly fucosylated samples were determined by mass spectrometry. Samples were partially reduced with 100 mm dithiothreitol in 100 mm Tris/HCl pH 8.0 at a concentration of 0.21 mg·mL^−1^. Samples were desalted online using a reversed‐phase cartridge prior to injection into the MS system (Agilent 6540 Q‐ToF equipped with Jetstream ESI source). Approximately 945 ng of each sample was loaded onto the column. The mass spectra of light and heavy chains were deconvoluted using maximum entropy algorithm.

### Covalent aggregates

An IgG1 sample after protein A purification was taken for the preparation of covalent aggregates. Five milliliters of IgG1 sample at 4 mg·mL^−1^ was placed at pH 3 for 1 h to create additional aggregates, followed by neutralization to pH 5 and a preconcentration on 30‐kD spin filters to > 100 mg·mL^−1^ and a final volume of ~ 75 μL. Fifty microliters of this high concentration sample was mixed with 2 μL 100 mm disuccinimidyl suberate (DSS) stock solution (Thermo Scientific) and incubated at room temperature for 15 min. The reaction was quenched with 2 μL 1 m Tris pH 7.8 and kept at room temperature for 15 min. Samples were diluted with 500 μL MQ water to a concentration of ~ 9 mg·mL^−1^. This sample was separated into fractions by preparative SEC.

### Preparative SEC purification

A preparative SEC purification was performed on the covalent aggregate sample and on the deamidated sample with elevated aggregate levels. A Superdex 200 10/30 column (24 mL) column was equilibrated with PBS pH 7.4 buffer using an ÄKTA explorer 100 system (GE life sciences) at a flow rate of 1 mL·min^−1^. An isocratic run in PBS pH 7.4 was performed at 0.75 mL·min^−1^ using 0.5 mL of each sample and fractions were collected based on UV 280‐nm signal. Collected fractions were analyzed on SDS/PAGE to determine the monomer, dimer, and higher oligomeric species in each fraction. Fractions with similar SDS/PAGE profiles were pooled for further analysis and are referred to as ‘covalent dimer’ or ‘covalent oligomer’.

### Low‐affinity Fcγ receptors relative binding determination

Recombinant human FcγRIIa, FcγRIIb, FcγRIIIa, and FcγRIIIb were biotinylated as previously reported 29. Fcγ receptors were then immobilized on a G‐Strep SensEye® sensor (Ssens BV) at 5 μg·mL^−1^ or 10 μg·mL^−1^ in 50 mm sodium acetate pH 4.5/0.05% Tween‐80 with a print time of 5 min.

Samples were analyzed in a relative binding approach on IBIS MX96 SPRi (IBIS Technologies BV, Enschede, the Netherlands) with HBS buffer pH 7.2/0.05% Tween‐80 as running buffer. A baseline of 1 min was followed by an association time of 2 min and a dissociation time of 1 min. Then, the sensor surface was regenerated with 25 mm phosphoric acid pH 3.0 in a single step of 30 s. Sensorgrams were referenced and zeroed. Binding levels at equilibrium (2 min) were used to determine relative binding levels. Relative binding was defined as the level of binding with respect to a reference sample, which is set to 100% binding activity. Relative binding was determined at 50 μg·mL^−1^ IgG1 and 250 μg·mL^−1^ IgG1 (FcγRIIa and FcγRIIIa) or 250 μg·mL^−1^ IgG1 and 1000 μg·mL^−1^ IgG1 (FcγRIIb and FcγRIIIb). Activity of Fcγ receptors at the sensor surface reduced over time, and we corrected for the decaying surface by applying a correction factor. Four calibration curves were injected distributed over the sample sequence. The decay in binding of these calibration curves was used to determine the correction factor for each sample, depending on the injection cycle number.

Specificity of the method was assessed by analysis of IgA samples with the same Fab region as the tested IgG samples, but on an IgA backbone instead of an IgG backbone. Both IgG references and IgA test samples were injected at concentrations of 3.33 μm and the binding of IgA samples at equilibrium was calculated relative to the binding of IgG samples at equilibrium, which were set at 100%.

### FcγRI kinetic determination

Single‐cycle kinetics of IgG1 on FcγRI was performed on a CAPchip (GE life sciences) with HBS‐EP+ as running buffer on a Biacore T200 instrument (GE Life Sciences). The CAPchip was used according to the manufacturers’ protocol. Recombinant FcγRI was captured on a CAPchip at 0.5 μg·mL^−1^ for 60 s at 2 μL·min^−1^. Five increasing sample concentrations of IgG1 were injected (0.06, 0.19, 0.56, 1.67, and 5 nm). The association time was set at 120 s, while the dissociation time at 900 s (flow rate 30 μL·min^−1^). Regeneration was performed for 60 s (flow rate 5 μL·min^−1^) according to CAPchip protocol. Analyses were performed at 37 °C. Data analysis was performed in the BiaEvaluation software (GE life sciences) and fitted to a 1 : 1 kinetic model to determine *k*_a_, *k*_d,_ and *K*_D observed_.

### FcRn kinetic determination

Multicycle kinetics of IgG1 on FcRn was performed on AR2G sensor tips in an Octet Red384 (Pall ForteBio, Portsmouth, UK). AR2G sensor tips were activated by 1‐ethyl‐3‐(3‐dimethylaminopropyl)carbodiimide hydrochloride (EDC)/*N*‐hydroxysuccinimide (NHS) according to the manufacturers’ protocol, followed by immobilization of 6 μg·mL^−1^ recombinant human FcRn in sodium acetate pH 5.0. After immobilization, the sensor tips were deactivated by ethanolamine pH 8 according to the manufacturers’ protocol.

IgG1s were analyzed in 50 mm phosphate/150 mm NaCl buffer/0.1% Tween 20 pH 6 at concentrations of 320 nm and 80 nm down to 2.5 nm. The samples at 320 nm were dissociated in 50 mm phosphate buffer/150 mm NaCl/0.1% Tween 20 pH 7.2, while dissociation for the remaining dilutions was performed in 50 mm phosphate/150 mm NaCl buffer/0.1% Tween 20 buffer pH 6. Regeneration of the sensor tips was performed with 100 mm Tris/HCl/200 mm NaCl/0.1% Tween 20 buffer pH 8. Data analysis was performed in corresponding software (Pall ForteBio), and sensorgrams were referenced and zeroed, followed by fitting to a heterogeneous ligand model to determine *k*_a_, *k*_d_ and *K*_D observed_ at pH 6. Additionally, the highest IgG concentration, dissociated at pH 7.2, was analyzed for *k*_d_ and fraction bound at 5 s after the start of dissociation. Fraction bound was determined at 5 s after the start of dissociation, with the response at *t* = 0 s after the start of dissociation was normalized to 100%.

### Fcγ receptor analysis on immobilized IgG1

Stressed IgG1 samples and reference samples were immobilized on a G‐COOH SensEye® sensor (Ssens BV) after activation with EDC/NHS according to the manufacturers’ protocol. Immobilization of the samples at 1 μg·mL^−1^ dilutions in 10 mm sodium acetate pH 4.5/0.05% Tween‐80 was performed in the continuous‐flow microspotter (CFM; Wasatch Microfluidics) using a print time of 5 min. Next, the sensor was deactivated with 1M ethanolamine pH 8.5 according to the manufacturers’ protocol.

Interaction measurements between monoclonal antibody and various recombinant human Fcγ receptors (FcγRI from R&D systems, Minneapolis, MN, USA, others from Synthon Biopharmaceuticals BV, Nijmegen, the Netherlands) were taken on an IBIS MX96 SPRi instrument (IBIS Technologies BV). Fcγ receptors were diluted into HBS buffer pH 7.2/0.05 w/v% Tween‐80 running buffer. The following start concentrations were used: FcγRI: 40 nm; FcγRIIa: 20 μm; FcγRIIb: 25 μm; FcγRIIIa: 20 μm; FcγRIIIb: 24 μm; and for each 8 twofold dilutions were made. A baseline of 2 min was followed by an association time of 5 min and dissociation at 1 μL·s^−1^ in 1 step for 4 min. The instrument was kept at 37 °C during analysis. Regeneration was performed with 25 mm phosphoric acid pH 3.0 in a single step of 30 s. Sensorgrams were referenced and zeroed, followed by steady‐state equilibrium affinity determination in Scrubber (BioLogic).

### Statistical data analysis

The results for each of the binding assays were statistically evaluated in Minitab. Duplicate or triplicate measurements were taken for each of the samples and methods. The relation between binding or affinity and the percentage of modification was determined with regression analysis.

## Results

### Assay development and method performance

**Figure 1:**
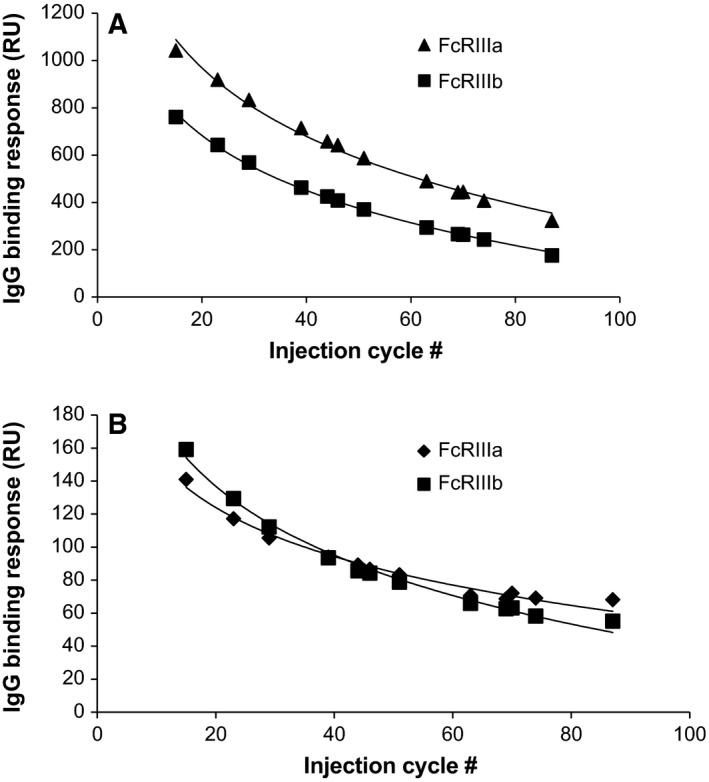
IgG binding response at 500 μg/mL to the four low‐affinity Fcγ receptors (A: FcγRIIIa and FcγRIIIb; B: FcγRIIa and FcγRIIb) during 90 sequential analyses. Each curve followed a logarithmic decay, which was used to correct for decay according to injection cycle number.

Low‐affinity Fcγ receptors were minimally biotinylated 29 followed by immobilization on a single streptavidin sensor. Degrees of labeling were between 0.3 and 0.5 for the different Fcγ receptors, and proper IgG binding was measured on each of the Fcγ receptors. Decay in IgG binding responses, indicative of receptor instability at the sensor surface, was measured. A 30–60% reduction in Rmax values was determined during 60 regeneration cycles. A regeneration buffer scouting as described in Geuijen *et al*. 30 showed that a regeneration buffer of 25 mm phosphoric acid adjusted to pH 3.0 was most suitable. Use of this regeneration buffer improved receptor stability, although still decay in binding was observed (Fig. 1). The fast on‐ and off‐rate of the receptors at the surface prevented the use of kinetic data. As the method was intended as a fast screening method, a relative binding approach was chosen.

The decay in IgG binding response to the receptor may be described by a logarithmic function (Fig. 1), which was used to correct for reduced binding. In an analytical run, four separate calibration curves of reference standard were injected distributed throughout the run, which were used to determine the values of the logarithmic function, with which the concentration of a sample at any cycle may be calculated. The validity of such a mathematical correction for the decay in response was verified in a method qualification, where range, accuracy, precision, specificity (Table 1), and model fit were assessed. Model fits to a logarithmic function had a *R* ^2^ > 0.995 in all experiments, and residuals were randomly distributed over the fitted curve. Specificity was assessed by injecting two different batches of IgA molecules, which should not bind to Fcγ receptors. Relative binding compared to an IgG reference was measured and was acceptable, although slightly higher values were measured on FcγRIIIb. Accuracy and precision data were analyzed in a general linear model in an ANOVA, and none of the parameters that were included (operator, run number, spot number) were significant factors that contributed to the variance. Due to decaying responses over time, sensor chips are freshly prepared in each new experiment, and therefore, no intermediate precision within chip preparation was included. Intermediate precision of test samples from the qualification was 12% or lower (Table 1), which is comparable to or below variability in binding studies based on kinetics (e.g., Katsamba *et al*. 31, Navratilova *et al*. 32, and Rich *et al*. 33), and therefore, variability was found acceptable for the intended purpose of the method.

**Table 1:** Range, accuracy, intermediate precision, and specificity of relative binding assay. LLOQ, lower limit of quantitation; LQC, low‐quality control; MQC, middle‐quality control; HQC, high‐quality control; ULOQ, upper limit of quantitation

Fcγ receptor	Range (μm)	Test sample	Nominal value (IgG; (μm)	Accuracy (%)	Intermediate precision (%)	Specificity: % binding of IgA
FcγRIIIa	0.104–3.33	LLOQ	0.1	96.6	7.75	
		LQC	0.2	97.6	5.59	0.5
		MQC	0.9	106.0	5.33	1.8
		HQC	2.7	84.4	8.08	
		ULOQ	3.1	93.8	5.13	
FcγRIIIb	0.832–26.67	LLOQ	0.9	110.6	4.48	
		LQC	3.1	87.1	2.70	9.9
		MQC	6.7	91.5	5.79	13.2
		HQC	20.0	104.4	7.12	
		ULOQ	22.2	104.3	11.99	
FcγRIIa	0.104–3.33	LLOQ	0.1	111.4	7.99	
		LQC	0.2	90.6	4.09	1.4
		MQC	0.9	92.0	7.50	3.3
		HQC	2.7	101.5	12.11	
		ULOQ	3.1	101.4	7.33	
FcγRIIb	0.832–26.67	LLOQ	0.9	106.2	5.93	
		LQC	3.1	90.3	3.64	14.3
		MQC	6.7	96.1	4.45	27.9
		HQC	20.0	101.5	6.81	
		ULOQ^a^	22.2	101.0	6.83	

a One sample was excluded due to an air bubble in the injection; *n* = 17. All other results are based on *n* = 18.

As previously mentioned, separate assays for FcγRI and FcRn were used. FcγRI interactions were measured in a single‐cycle kinetics measurement where five dilutions of IgG were injected on captured biotinylated FcγRI with an intermediate precision in *K*_D_ of 9.6%. A multicycle kinetics measurement based on BLI was developed for FcRn, where association was performed at pH 6 and dissociation of the highest IgG concentration was performed at pH 7.4 and dissociation of the other IgG concentrations measured at pH 6. Intermediate precision of 11.0% on *K*_D_ and 14.0% on fraction bound at neutral pH was determined. Method performance of both methods was found acceptable. Sample throughput of the FcγRIIa/b/FcγRIIIa/b and FcRn methods was high, with only 5‐min analysis time per sample. Unfortunately, the throughput of the FcγRI method was somewhat lower compared to the other two methods, with 45 min per sample but still acceptable for the high‐throughput screening purpose of this study. In the end, three separate screening methods for full Fc tail functionality of IgGs were available which all passed the set qualification criteria.

### Characterization of stressed samples

A selection of the most common degradations in IgGs was made to measure the impact on Fc effector function, by studying binding to Fc receptors on the three screening assays. IgG1 samples were subjected to accelerated Ox, accelerated deamidation, thermal/shake stress, FT, and deglycosylation (DG). Additionally, a few IgG samples with aberrant/different fucosylation levels were available for Fc effector binding, induced by applying variations in bioreactor process parameters. The stressed IgG samples were modified at the level of Ox (mainly H:Met252), D‐N (three main sites in this IgG1), aggregation levels, and the percentages of DG. These IgG samples were analyzed for antigen target binding by SPR or ELISA in case of aberrantly fucosylated samples (Table 2). Peptide mapping‐based methods were used to quantify the levels of Ox and deamidation and HP‐SEC to determine the aggregate levels (Table 2). Next, the IgG samples were analyzed on SPR and BLI to measure the binding to Fc receptors (Table 3).

**Table 2:** Results of reference analyses to determine stress levels and antigen target binding. n.d., not determined. Ox, oxidation; D‐N, aspargine deamidation; F‐T, freeze‐thaw cycles; DG, deglycosylation; AF, afucosylation, HC, heavy chain; LC, light chain; CDR, complementary‐determining region; Fc, fragment crystallizable; HMW, high molecular weight species

Stress condition	Ox Met255 (%)	D‐N HC CDR (%)	D‐N LC CDR (%)	D‐N HC Fc (%)	HMW (%)	Insoluble HMW (%)	Deglycosylatio sn (%)	AF (%)	Antigen target binding (%)
Reference	2.5	9.8	9.7	3.8	1.3	n.d.	n.d.	11	100.0
H_2_O_2__0.1%	3.7	9.6	9.6	n.d.	1.2	n.d.	n.d.	11	100.9
H_2_O_2__0.25%	5.1	9.6	9.7	n.d.	1.3	n.d.	n.d.	11	100.7
H_2_O_2__0.5%	7.1	9.6	9.6	n.d.	1.3	n.d.	n.d.	11	99.3
pH7.2_48 h	2.8	10.1	13.5	5.7	1.7	n.d.	n.d.	11	97.6
pH7.2_72 h	2.9	10.0	15.3	5.3	2.1	n.d.	n.d.	11	96.5
pH7.2_96 h	2.9	10.4	17.4	5.1	2.3	n.d.	n.d.	11	94.9
pH8.0_48 h	3.2	12.5	27.5	9.4	2.3	n.d.	n.d.	11	85.3
pH8.0_72 h	3.4	13.7	34.6	10.1	2.2	n.d.	n.d.	11	80.9
pH8.0_96 h	3.7	15.7	40.6	10.6	4.5	n.d.	n.d.	11	75.5
40 °C‐24 h	n.d.	n.d.	n.d.	n.d.	1.6	n.d.	n.d.	11	96.4
40 °C‐48 h	n.d.	n.d.	n.d.	n.d.	2.0	n.d.	n.d.	11	95.2
40 °C‐72 h	n.d.	n.d.	n.d.	n.d.	2.0	n.d.	n.d.	11	94.1
70 °C_15 m	n.d.	n.d.	n.d.	n.d.	1.7	1.15	n.d.	11	103.0
75 °C_15 m	n.d.	n.d.	n.d.	n.d.	1.5	51.4	n.d.	11	61.9
F‐T 1	n.d.	n.d.	n.d.	n.d.	1.2	n.d.	n.d.	11	101.9
F‐T 5	n.d.	n.d.	n.d.	n.d.	1.2	n.d.	n.d.	11	100.4
F‐T 10	n.d.	n.d.	n.d.	n.d.	1.3	n.d.	n.d.	11	101.6
DG 0	n.d.	n.d.	n.d.	n.d.	n.d.	n.d.	0	11	92.2
DG 100	n.d.	n.d.	n.d.	n.d.	n.d.	n.d.	100	11	95.9
AF 3	n.d.	n.d.	n.d.	n.d.	n.d.	n.d.	n.d.	3	103.1
AF 8	n.d.	n.d.	n.d.	n.d.	n.d.	n.d.	n.d.	8	100.8
AF 70	n.d.	n.d.	n.d.	n.d.	n.d.	n.d.	n.d.	70	115.6

**Table 3:** Summarized results of stressed IgG1 samples on Fc receptor binding. n.d., not determined. F‐T, freeze‐thaw cycles; DG, deglycosylation; AF, afucosylation

Stress condition	Relative binding (%)				Affinity (nm)		Fraction bound (%)
	FcγRIIIa	FcγRIIIb	FcγRIIa	FcγRIIb	FcγRI	FcRn	FcRn
Reference	100.0	100.0	100.0	100.0	0.56	5.7	8.3
H_2_O_2__0.1%	103.6	93.1	103.9	107.4	0.93	n.d.	n.d.
H_2_O_2__0.25%	102.8	101.1	104.5	103.5	0.53	6.2	6.4
H_2_O_2__0.5%	97.4	94.9	100.1	100.1	0.42	6.2	7.7
pH7.2_48 h	97.4	89.6	140.3	92.9	0.59	5.5	7.6
pH7.2_72 h	92.7	86.5	143.9	92.9	0.48	6.1	8.3
pH7.2_96 h	96.9	88.3	132.1	94.7	0.58	5.6	7.9
pH8.0_48 h	59.5	52.5	76.6	54.4	0.52	6.0	7.8
pH8.0_72 h	53.2	53.8	64.5	53.6	0.55	5.5	6.5
pH8.0_96 h	61.8	65.9	78.2	61.8	0.55	5.3	10.0
40 °C‐1000 r.p.m._24 h	96.8	106.1	92.5	101.1	0.53	5.5	7.9
40 °C‐1000 r.p.m._48 h	101.8	105.9	91.8	100.1	0.45	5.0	7.3
40 °C‐1000 r.p.m._72 h	102.9	107.1	97.5	103.1	0.53	5.4	8.6
70 °C_15 m	72.7	126.7	62.5	93.4	n.d.	34.2^a^	n.d.
75 °C_15 m	388.7	> 1000	> 1000	> 1000	n.d.	0.4^a^	n.d.
F‐T 1	80.1	106.5	102.2	102.3	0.52	5.7	8.4
F‐T 5	103.0	104.7	92.3	97.2	0.42	5.7	8.1
F‐T 10	98.7	102.0	85.9	95.9	0.48	5.2	7.4
DG 0	106.2	104.9	99.5	105.3	0.52	4.9	8.5
DG 100	6.5	10.5	10.3	15.3	No fit	6.3	4.1
AF 3	79.7	83.9	101.5	90.6	0.44	3.7	14.3
AF 8	87.7	93.0	97.6	84.2	0.43	3.7	14.1
AF 70	158.6	186.3	103.5	110.5	0.33	3.8	15.7

a Nonspecific binding to reference channels was measured; kinetics on FcRn could not be determined.

D‐N was measured on all potential deamidation sites, and three major sites were detected. Two deamidation sites are present in the CDR of the antibody [referred to as heavy chain complementary‐determining region (HC‐CDR) and light chain complementary‐determining region (LC‐CDR), which refers to heavy chain and LC‐CDR regions, respectively] and one site is present in the Fc region of the antibody [referred to as HC‐Fc (heavy chain Fc region)]. Deamidation levels increased up to ~ 15% and 40% for the two sites in the CDR, respectively. In the Fc region, D‐N increased up to 10%. High molecular weight (HMW) species increased during forced deamidation for 96 h to 4.5%, which may influence the measurements, and this was further investigated by separating HMW species from monomer (see below). Increased HMW species were also detected in the samples that were heated to 70 and 75 °C. In all other stressed IgG samples, the levels of HMW species remained similar to the references. Ox levels in IgG samples that were exposed to H_2_O_2_ increased to ~ 7%. Antigen target binding remained unaffected under the applied stress conditions, except for (1) the deamidated IgG samples due to two deamidation sites that are present in the CDR and (2) thermal/shake‐stressed IgG samples at 75 °C/300 r.p.m. for 15 min.

No altered binding to any of the Fc receptors was measured for the IgG samples that were subjected to thermal/shake stress and FT (Table 3), and therefore, these results are not further discussed.

### Low‐affinity Fc receptors screening

Binding to the low‐affinity Fcγ receptors (FcγRIIIa, FcγRIIIb, FcγRIIa, and FcγRIIb) was measured in a relative binding setup, where a reference standard was set to 100% activity and stressed IgG samples were measured relative to this standard.

**Figure 2:**
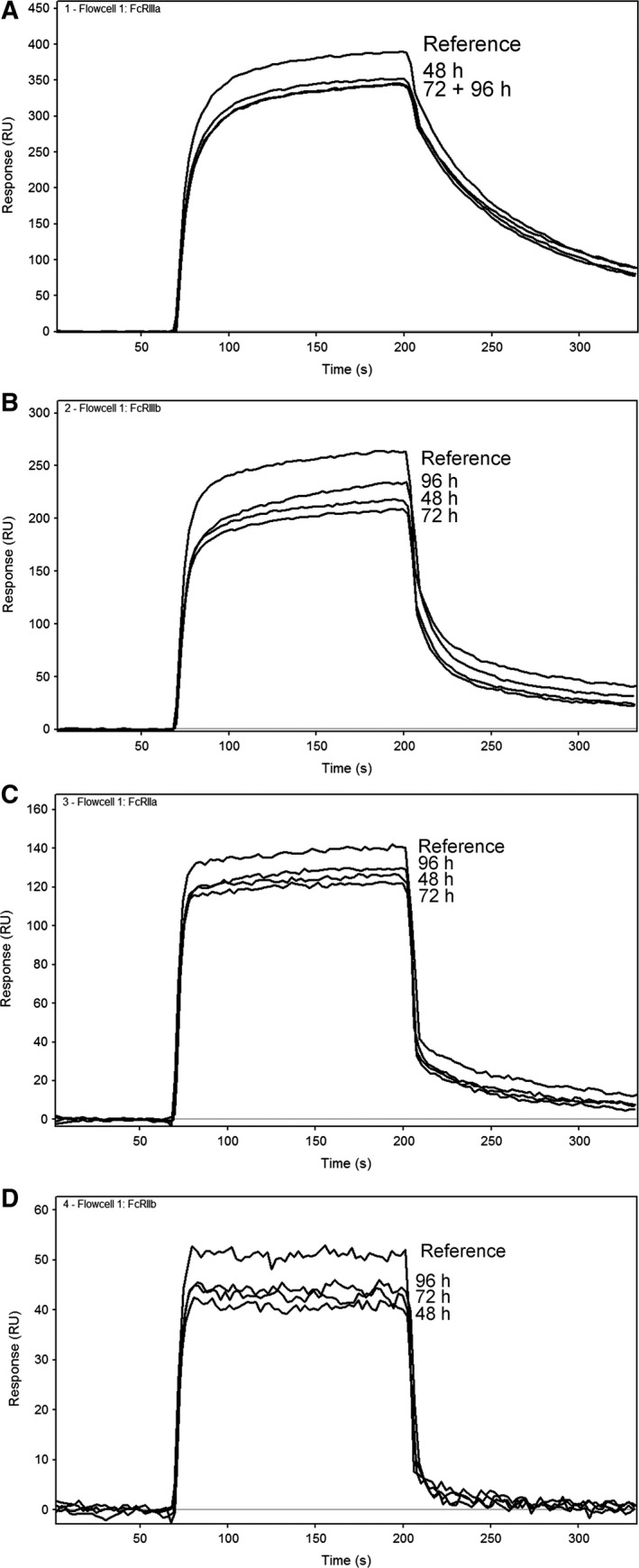
Sensorgrams of a reference IgG sample and the deamidated IgG samples at pH 8 at different time points. Injections at 250 μg·mL^−1^ IgG are shown on (A) FcγRIIIa, (B) FcγRIIIb, (C) FcγRIIa, and (D) FcγRIIb, respectively.

Asparagine deamidation reduced binding to the low‐affinity Fcγ receptors to ~ 50–80% binding relative to the reference (Table 3 and Fig. 2). A regression analysis of the percentage of deamidation against relative binding showed that these differences are statistically significant (*P* < 0.0005 for each of the Fcγ receptors). An increase in HMW species was observed in the deamidated samples next to increased deamidation levels. The lower relative binding on the low‐affinity Fcγ receptors of these samples may therefore also be induced by aggregates. The deamidated sample (72 h) was separated into a monomer and aggregated fraction by preparative SEC. The monomeric deamidated peak was analyzed on analytical SEC and was found to be pure monomer directly after separation. After short‐term overnight storage, the sample contained ~ 0.5% HMW species, which cannot be avoided due to the intrinsic property of IgGs to form a small fraction of aggregates (Fig. S1). However, this low aggregate level is comparable to the reference sample, and therefore, this sample was considered a representative sample to study the effect of deamidation only. Measurement of relative binding to FcγRIIIa/b and FcγRIIa/b resulted in ~ 50–80% relative binding of the monomeric deamidated sample, compared to ~ 60% of the nonpurified sample (Fig. S2). These data indicate that deamidation alone reduces binding to Fcγ receptors.

No difference in binding was observed after oxidative stress (Table 3), which is generally found in the literature as well 14, 20. Only Bertolotti‐Ciarlet *et al*. 20 found a decrease in binding (roughly 20% decrease) on FcγRIIa with IgG oxidized on Met252 to 80%. However, the methionine Ox levels in our stressed samples did not exceed 7% of Ox, which may explain this difference in results.

Lack of binding of deglycosylated IgG to the low‐affinity Fcγ receptors has been described extensively 12, 13, 14, 15, 16 and was confirmed in our study. Fully deglycosylated IgG1 had a maximum of 15% binding response relative to the reference (*P* < 0.0005 for all low‐affinity Fcγ receptors). The glycans in the Fc region of an antibody have a stabilizing (i.e., IgG folding) effect and are required for a proper interaction with these Fcγ receptors 34.

Apart from full DG of the IgG1, we analyzed IgG1s with aberrant/different fucosylation levels. A different feed strategy in the bioreactor was applied, which resulted in IgGs with afucosylation (AF) levels of 3%, 8%, and 70%, respectively. Variation in AF only affected binding to FcγRIIIa and FcγRIIIb; a significant difference in relative binding between the three samples is measured from low to high corresponding to the AF levels (*P* < 0.0005 in regression analysis for both receptors). A binding of 158–186% on FcγRIIIa and FcγRIIIb of the sample that was afucosylated for 70% was measured relative to the reference. The 3% and 8% afucosylated samples had a relative binding of 80–93%, which is due to the slightly lower AF level compared to the reference sample (11% AF). Binding to FcγRIIa and FcγRIIb was unaffected by the lower AF levels (Table 3).

### FcγRI

**Figure 3:**
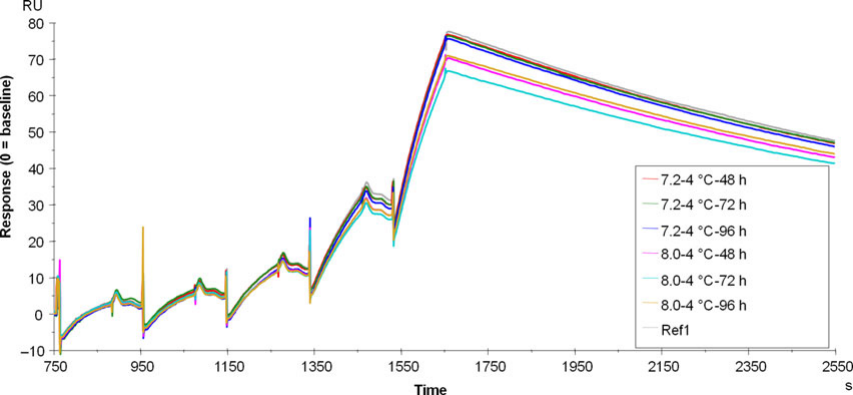
Overlay of single‐cycle kinetics sensorgrams of deamidated and control IgG samples on FcγRI binding.

The high‐affinity interactions on FcγRI were measured in a single‐cycle kinetic determination at pH 7.4. To our knowledge, no literature is available that describes the effect of IgG deamidation and FcγRI binding. We found no effect of deamidation of IgG1 on binding to FcγRI as shown in Fig. 3. No effect of Ox or fucosylation degree of IgG was measured on FcγRI binding, confirming earlier results in the literature on FcγRI binding for oxidized IgG1 20 and fucosylation 17, 18.

**Figure 4:**
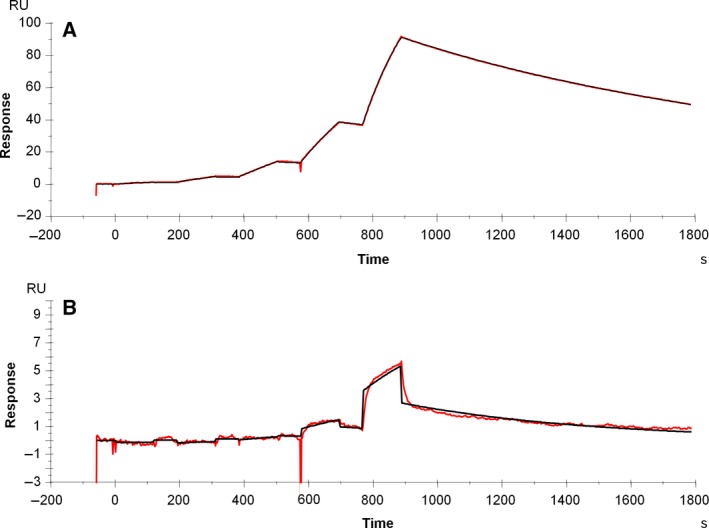
Single‐cycle kinetics sensorgrams of glycosylated (A) and deglycosylated (B) IgG samples on FcγRI binding. Measured sensorgrams are shown in red and fitted curves shown in black.

Deglycosylated IgG almost completely prevented binding to FcγRI (Fig. 4), as shown by the maximum response of ~ 5 resonance units (RU) compared to 80 RU of the reference sample. A 1 : 1 kinetic fit was applied to the sensorgrams, which resulted in poor fits of the fully deglycosylated sample. The resulting kinetic parameters cannot be reliably determined and are not reported. Our results do not fully confirm earlier findings in the literature, as ~ 60% binding to FcγRI remained in earlier studies 12, 13. Hence, we investigated binding of FcγRI to deglycosylated IgG1 by inverting the experimental setup. Deglycosylated and glycosylated IgG1 were immobilized on the sensor surface and their binding to FcγRI in solution was analyzed. Virtually, no binding to FcγRI was found (Fig. S3E), confirming our results as presented in Fig. 4.

### FcRn

The mechanism of FcRn‐mediated IgG recycling is complex and encompasses IgG association at pH 6 and dissociation at pH 6 and pH 7.4. Most cited references only studied kinetics on FcRn at pH 6. Here, FcRn interactions were measured in a multicycle kinetics experiment of eight IgG1 dilutions. The lowest seven dilutions were used for kinetics determination at pH 6 by fitting both the association and dissociation phase to a heterogeneous ligand model, as proposed by Vaughn and Bjorkman 35. The highest IgG dilution was associated at pH 6, and dissociation was measured at neutral pH. The dissociation rate and fraction bound at neutral pH were determined from this injection only.

**Figure 5:**
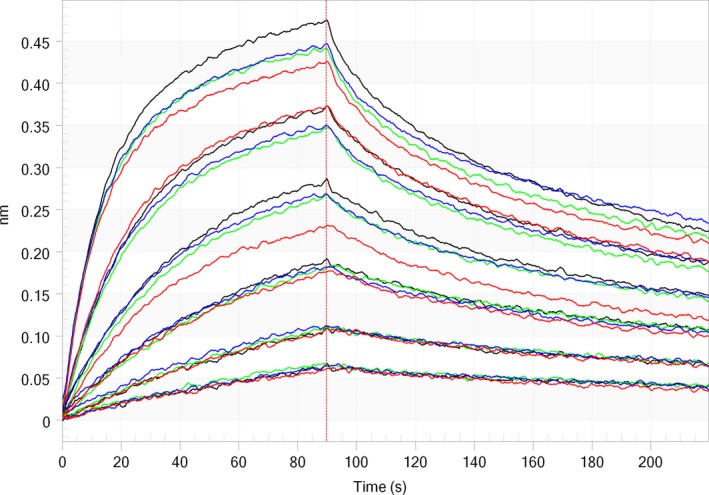
Overlay of sensorgrams of deamidated samples on FcRn binding (reference in black; *t* = 48 h/pH 8 in green; *t* = 72 h/pH 8 in red; and *t* = 96 h/pH 8 in blue). IgG concentrations between 2.5 and 10 nm.

No effect of IgG on FcRn binding at pH 6 was measured after deamidation in our assay, and no differences in dissociation and fraction bound at neutral pH were measured (Fig. 5). FcRn affinity and the fraction bound at neutral pH did not change depending on the fucosylation levels (Table 3).

**Figure 6:**
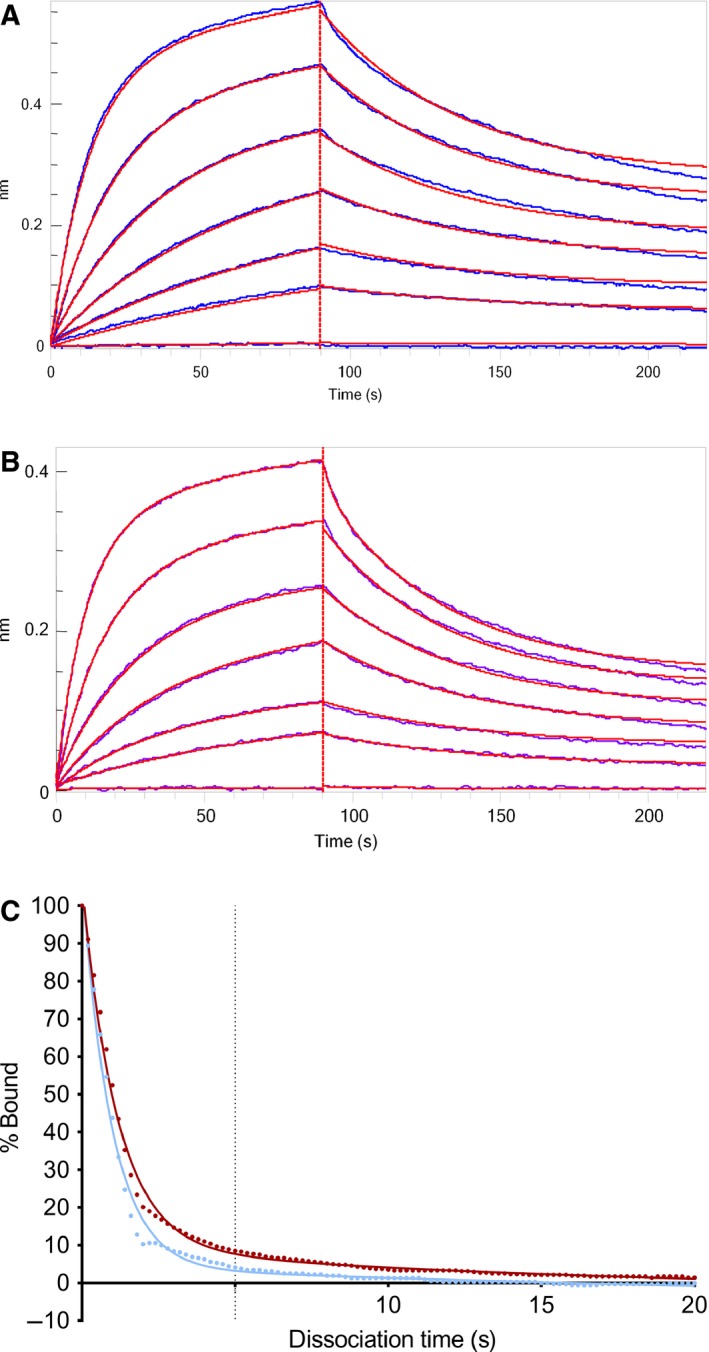
Sensorgrams of IgG1 binding to FcRn of glycosylated (A) and deglycosylated (B) IgG1. Fitted curves are shown in red. (C) The fraction bound at neutral pH of glycosylated (red) and deglycosylated (blue) IgG.

Deglycosylation resulted in a minor reduction in FcRn binding in a linear regression analysis (*P* = 0.005). Additionally, measurements at neutral pH indicate a significantly lower fraction bound and a faster dissociation rate after DG (Fig. 6 and Table 3). Deglycosylated IgG is still able to bind to FcRn, but dissociation at neutral pH is faster compared to the glycosylated counterpart, which may be important for the serum half‐life.

We did not measure a significant decrease in affinity at pH 6 or in fraction bound at neutral pH on FcRn after methionine Ox, whereas other publications 20, 21, 22, 23 indicate that FcRn binding is reduced upon methionine Ox (only studied at pH 6). However, highest Ox levels that we induced were around 7%, whereas other groups report differences in FcRn binding at levels close to 80% of methionine Ox. Stracke *et al*. 21 found that only one of the two heavy chains is oxidized when Ox levels are around 50% or lower, and the other heavy chain of the antibody is still able to bind to FcRn. This agrees well with our results as no impact is measured at 7% Ox.

### Presence of high molecular weight species

**Figure 7:**
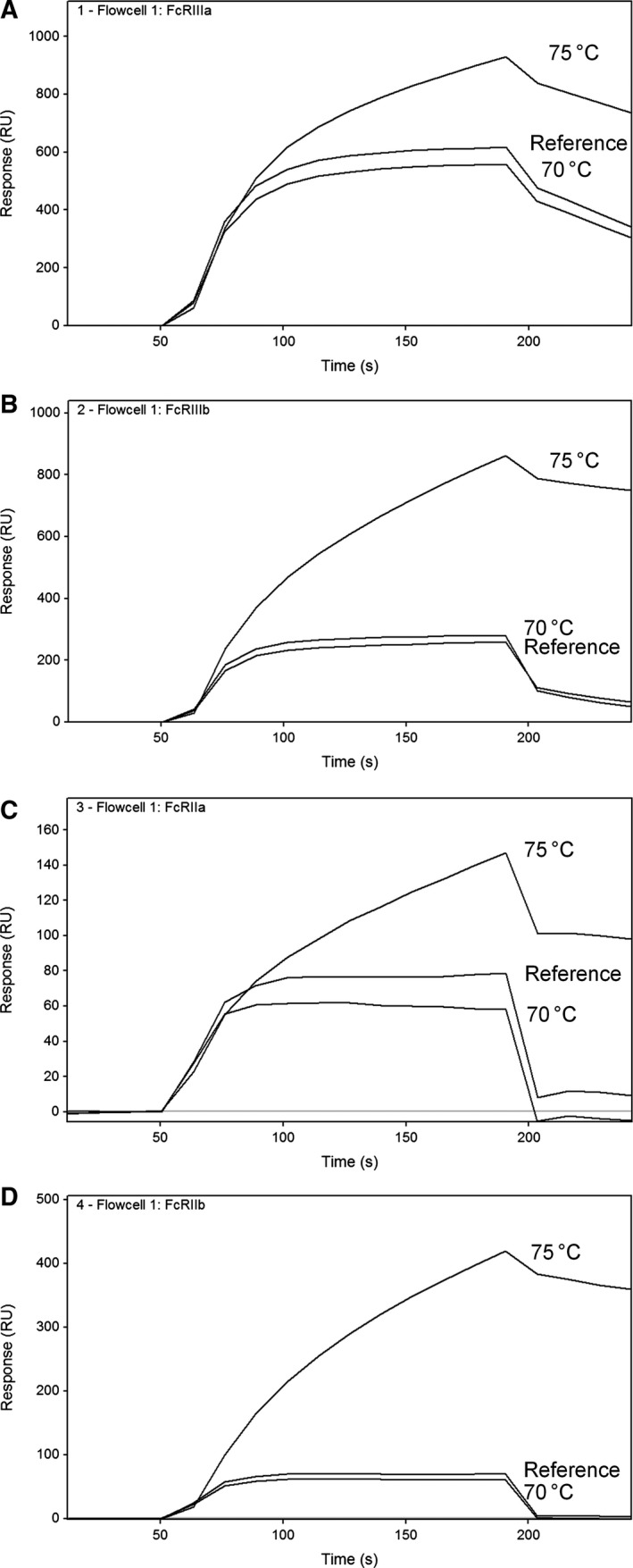
Sensorgrams (measured at 250 μg·mL^−1^) of reference and aggregated IgG samples heated to 70 and 75 °C for 15 min on FcγRIIIa (A), FcγRIIIb (B), FcγRIIa (C), and FcγRIIb (D), respectively.

As mentioned above, the presence of aggregates in our stressed IgG samples could impact the binding to the Fcγ receptors and FcRn. Previous studies have already emphasized the importance to control the level of aggregates during these types of binding studies 15, 24, 25. This was observed in deamidated IgG samples where the fraction of HMW species increased with a few percent. Additionally, samples were heated to 70 and 75 °C to induce larger fractions of HMW species. HMW species impact binding to all of the Fc receptors. IgG samples were heated for 15 min at temperatures close to the first T_m_ (melting temperature) of the IgG1, which resulted in differential binding to the low‐affinity Fcγ receptors (Fig. 7). Heating to 70 °C, which is just below the T_m_, decreased the relative binding to the low‐affinity receptors, except for FcγRIIIb where an increase was observed. However, heating to 75 °C resulted in at least 4× more binding (relative binding 400% or higher), likely due to avidity effects of large aggregates that were present in these samples (Fig. 7).

Heating of the IgG samples, especially to 75 °C, resulted in a large fraction of insoluble aggregates, which behave completely different from monomers in our binding assay. A more controlled approach for aggregate preparation was performed by covalent coupling of IgG1s to each other using a chemical linker. Preparative SEC was used to separate the monomer from dimers, trimers, and higher aggregates as described in Materials and methods (Fig. S4). The covalent dimers and oligomers that were separated by SEC showed similar behavior in the relative binding measurement on low‐affinity Fcγ receptors compared to the heated samples (results not shown). Relative binding up to 400–800% on each of the low‐affinity Fcγ receptors was measured.

Furthermore, the covalent dimers were analyzed in the FcγRI and FcRn binding assays. FcγRI binding with dimeric samples resulted in an apparent slower off‐rate (Fig. S5). and as a consequence, an apparent higher affinity is measured with the covalent aggregate samples (Table 4). In case of FcRn, kinetic evaluation of the binding curves results in a 1 : 1 binding model at pH 6 for the dimer/oligomer sample, whereas the monomeric samples were fitted with a heterogenous ligand model (Fig. S6). The dimeric sample could be equally well fitted with a 1 : 1 binding model and a heterogeneous ligand model. We have chosen to fit the 1 : 1 binding model for this sample. A difference in observed *K*_D_ and fraction bound at neutral pH was measured between monomer and dimer or oligomer samples (Table 4). However, the curve fitting was not corrected for a difference in molecular weight of the complex, because these were a mixture of monomers, dimers, and trimers and no actual molecular mass could be determined. Assuming a molecular weight of 300 kDa for dimers instead of 150 kDa still resulted in an equally good fit with the 1 : 1 binding model and the heterogeneous ligand fit, still with different kinetic parameters compared to the monomeric reference.

**Table 4:** Comparison of the effect of aggregate levels in IgG1 samples with respect to FcγRI and FcRn binding. N.d., not detected

	% Dimers	% Trimers and higher	*K*_D_ (nm) FcγRI	*K*_D_ (nm) FcRn	Fraction bound (%) FcRn
IgG1 reference	1.2	n.d.	0.66	6.3	7.5
Monomer IgG1	1.8	n.d.	0.52	7.0	8.9
Dimer IgG1	76.7	5.1	0.08	3.1^a^	17.3
Oligomer IgG1	73.4	14.0	0.07	3.0^a^	17.7

a 1 : 1 binding model applied instead of heterogeneous ligand model.

The purified covalent aggregates contained ~ 73–77% dimers and 5–14% trimers and higher oligomers, which resulted in a six‐ to eightfold increase in apparent affinity on FcγRI and twofold increase in apparent affinity and fraction bound on FcRn. The increased apparent affinity is most likely an avidity effect than a true difference in affinity.

As an additional verification of the results, we measured all FcR interactions in the opposite setup, where we immobilized the various stressed samples on a single SPR sensor and analyzed the binding to the different Fcγ receptors subsequently. In this setup, no differences in aggregated samples compared to the references were measured (Fig. S2). Affinities that were determined on FcγRI in the opposite setup matched closely to affinities that were measured for the aggregated samples in solution (0.2 nm for immobilized IgG vs 0.08 nm for dimeric IgG in solution), whereas the monomeric IgG in solution has an affinity of ~ 0.56 nm under tested conditions. Upon immobilization of the IgGs onto the sensor surface, pseudoaggregates are created when the IgG molecules are immobilized in close proximity to each other, and this may mask the differences that are caused by actual aggregates.

## Discussion

We assessed Fc tail functionality of IgG1 after exposure to various stress conditions using binding assays. Stress conditions that were applied and that did impact Fc tail functionality included D‐N, DG, aberrant fucosylation, or aggregation (Table 5). Importantly, no effects were measured after methionine Ox, thermal/shake stress, or repeated FT. Furthermore, we determined FcRn binding at pH 6 (kinetics) and at neutral pH (dissociation rate and fraction bound). Dissociation at neutral pH may be an important predictor for serum half‐life of antibodies 9, 36; however, most publications described the binding to FcRn at pH 6 alone. Instead, dissociation at pH 7.4 after association at pH 6 was analyzed here, resulting in a faster dissociation and lower fraction bound at pH 7.4 for a deglycosylated IgG sample. Other stress conditions did not influence FcRn dissociation at pH 7.4.

**Table 5:** Summary of Fc tail interactions to monitor for changes in product characteristics

IgG modification	FcγRIIIa	FcγRIIIb	FcγRIIa	FcγRIIb	FcγRI	FcRn
Deamidation (10–50%)	**Reduced relative binding**	**Reduced relative binding**	**Reduced relative binding**	**Reduced relative binding**	**No impact**	**No impact**
DG (100%)	Hardly any binding	Hardly any binding	Hardly any binding	Hardly any binding	Hardly any binding	**Slightly faster off‐rate. Lower fraction bound at neutral pH**
Aberrant fucosylation (3–70%)	Increased binding with lower fucosylation	Increased binding with lower fucosylation	No impact	No impact	No impact	**No impact**
Aggregation (5–75%)	Higher relative binding (> 400%)	Higher relative binding (> 400%)	Higher relative binding (> 400%)	**Higher relative binding (> 400%)**	Slower off‐rate, increased *K*_D_	Slower off‐rate, increased *K*_D_, 1 : 1 binding model
Ox (< 7% on Met^252^)	No impact	No impact	No impact	No impact	No impact	No impact
Thermal/shake stress	**No impact**	**No impact**	**No impact**	**No impact**	**No impact**	**No impact**
F/T	**No impact**	**No impact**	**No impact**	**No impact**	**No impact**	**No impact**

Results in boldface indicate results that have not been reported in the literature to authors’ knowledge.

The impact of D‐N of IgG on FcRn binding was previously reported by Gandhi *et al*. 27, and no impact of deamidation on FcRn binding at pH 6 was found. Here, no impact of deamidation on FcγRI (pH 7.4) and FcRn (both pH 6 and pH 7.4) was measured. On the other hand, relative binding on the low‐affinity Fcγ receptors was reduced after D‐N (50–70% of reference). Upon deamidation, also the percentage of HMW species increased, and therefore, the deamidated sample was purified into a monomeric fraction. In the purified monomeric deamidated sample, reduced binding was still measured on the low‐affinity Fcγ receptors, which could only be attributed to D‐N. The main deamidation site of this IgG is present in the CDR at the LC (up to 40% modified), which is positioned relatively far away from the Fc interaction site (lower hinge, upper CH2 domain 37, 38). Deamidation at this position is not likely to change the folding of the protein in such a way that it would have a large impact on Fc receptor binding. 3D models of both structures do not point in the direction of altered Fc binding induced by CDR deamidation (Fig. S7). In the lower hinge and upper CH2 domain, no potential deamidation sites are present. In the Fc region (CH2 and CH3 domains), other deamidation sites are present, which are less vulnerable toward deamidation, but are affected after stress conditions. The major deamidation site in the Fc region of the heavy chain (amino acid sequence SNGQPENNY) was deamidated at levels around 10%. Shields *et al*. 11 have studied binding behavior on all Fc receptors by point mutation of amino acids in the Fc region and did not find any influence of the amino acids in this deamidation site (altered binding defined as reduction of 40% or more). Here, the reduced binding of the deamidated samples was 30–50%. After all three incubations (48, 72, and 96 h), relatively similar binding levels and deamidation levels (around 10%) were found, whereas deamidation levels on the other two main deamidation sites in the CDR steadily increased over time. Collectively, this suggests that the HC‐Fc deamidation is most likely responsible for reduced binding to low‐affinity Fcγ receptors after deamidation. Asparagine residues sensitive toward deamidation may differ between different IgGs as these may be present in the CDR region and can therefore be specific toward the studied antibody. However, our results suggest that the major deamidation site which affects Fc receptor binding is present in the conserved residues of the Fc region (SNGQPENNY). These results indicate, together with data from Shields *et al*. 11, that the effects of deamidation on Fc receptor binding are not IgG dependent.

The structure of an IgG, with two heavy chains that both can potentially bind to Fc receptors, complicates analysis of these molecules. Fc tail interactions are not necessarily impacted by modifications on one of the heavy chains alone. If only one heavy chain is involved in an interaction and one heavy chain remains unaffected, this does not necessarily impact Fc effector binding, as shown for methionine Ox. In the Fc region of an IgG, two main Ox sites (H252 and H428) are present, of which H252 is the most vulnerable Ox site. Houde *et al*. 14 found that the conformation of IgGs is changed upon methionine Ox, although this is not reflected in an altered binding to FcγRIIIa, which may be a result of one Fc tail that can still bind to the Fcγ receptor. No difference in relative binding of IgG on FcγRIIIa, neither on any of the other low‐affinity Fcγ receptors, was measured with Ox levels up to 7% after H_2_O_2_ stress in our study. Furthermore, no differences in affinity and kinetics of oxidized IgG to FcγRI or FcRn (pH 6 and pH 7.4) were detected. This is in agreement with results published by Bertolotti‐Ciarlet *et al*. 20 who studied the interaction of IgGs with each of the Fcγ receptors. A few publications described the effect of methionine Ox on FcRn binding measured at pH 6 21, 39. In these studies, it was demonstrated that a single Met252 Ox (i.e., one heavy chain modified) has no impact on FcRn binding kinetics. IgG with both heavy chains oxidized alter the binding kinetics to FcRn significantly, resulting in faster plasma clearance. However, these measurements were only taken at pH 6. Therefore, we additionally measured dissociation rate and fraction bound at neutral pH and no differences in FcRn binding with Ox levels up to 7% were shown. The average methionine Ox of the studied IgG during production and processing did not exceed 2–3%. Hence, no impact on Fc tail functionality was expected. Wang *et al*. 39 analyzed IgG samples with a shelf life of 3 years under refrigerated or frozen conditions. Even then, IgG Ox levels did not exceed 13% and no effect on FcRn binding at pH 6 was detected. In summary, we postulate that both heavy chains should be oxidized in order to affect Fc tail functionality.

Hardly any IgG binding to the low‐affinity Fcγ receptors and FcγRI was measured after DG, which is in agreement with results from others 12, 13, 15, 40. The binding to FcRn receptor is not or only moderately influenced by the glycan occupancy, as similar affinity at pH 6 was measured using deglycosylated IgG1 compared to the glycosylated reference IgG. However, dissociation at neutral pH was impacted by glycan occupancy, as the fraction bound at neutral pH significantly decreased after DG. Furthermore, fucosylation levels of the antibody have an impact on the binding to FcγRIIIa and FcγRIIIb, whereas no differences in binding were measured on any of the other Fc receptors. These results are in agreement with the literature 13, 16, 17, 18, 19. None of the cited references studied the effect of DG on fraction bound at neutral pH, and we have demonstrated that there is a significant impact. A decrease in fucosylation induces stronger binding to FcγRIIIa and as such increases the ADCC of the antibody. This increased affinity is caused by carbohydrate–carbohydrate interactions of both the IgG and the Fcγ receptor 19. IgG glycosylation is important as it adds to the stability of the protein 41 and to maintain its effector binding characteristics, 12 both in glycan site occupancy and in glycosylation pattern differences (e.g., fucosylation levels). IgGs are more prone to aggregation when glycans are absent, which in turn has an effect on Fc effector functions. Furthermore, glycans stabilize IgGs against proteases that may cleave the protein during harvesting or purification, and as such, proper glycan occupancy is critical for the quality of a therapeutic antibody, especially when effector functions of the immune system are involved in the mode of action 40, 42.

The results for each of the Fcγ receptors indicate that dimers and oligomers, or aggregates, of IgGs bind stronger to the various types of Fc receptors and can therefore have a significant impact on affinity determinations. The binding of dimeric and oligomeric IgGs to low‐affinity Fcγ receptors changes, due to avidity effects, and is reflected in an increase in relative binding to 400% or higher. Comparable increased affinities have been measured by Luo *et al*. 24 Similarly, Bajardi‐Taccioli *et al*. 23 demonstrated an increase in relative activity on an FcRn binding assay when aggregates were spiked into the measured samples. A slower off‐rate was measured with samples that contained up to 86% of aggregation. These results were all obtained with samples that contained a significant amount of aggregates (more than 80%). On the other hand, samples that contain no more than 2.5% of aggregates have no altered relative binding in our study, whereas Dorion‐Thibaudeau *et al*. 15 found that HMW levels of only 2% already affected the binding to FcγRIIIa in their assay. Due to the avidity effects of aggregates, the impact of a small fraction of dimers and higher oligomers in samples can alter binding to Fc receptors and can therefore not be neglected. Protein aggregates may consist of reversible and irreversible aggregates 43. Aggregates that are artificially created (heating or chemically coupling) can generally be well characterized by other analytical assays 26, 43, 44, whereas reversible aggregates of IgGs which naturally occur may fall apart upon dilution 43 and are therefore difficult to characterize. The nature of aggregates in stressed IgG samples may be different compared to naturally occurring aggregates, which complicates the assignment of the impact these have in binding assays. Still, we strongly recommend controlling the aggregate level of samples when assessing Fc interactions in binding assays such as those described here.

No difference in binding was observed when aggregates were immobilized on the sensor surface. Most likely, the effects of aggregation are masked upon immobilization of IgGs in close proximity to each other. The immobilization of IgGs on the surface can cause the IgGs to behave as aggregates rather than monomeric molecules as they are covalently linked to the sensor surface in close proximity to each other, and therefore, the differences between monomer and aggregate are no longer measured.

Clinical relevance of the changes found is difficult to predict. For FcRn, the most prominent changes are to the fraction bound at neutral pH. Wang *et al*. 9 found an approximately threefold span in terminal half‐life differences for mAbs with reported fractions bound between ~ 0% and 15%. The differences found in this study are within an approximate twofold difference to the reference monomer. FcRn binding in itself may have limited predictive value on half‐life differences between IgGs and should be approached in a holistic fashion 10. Clinical impact of monoclonal antibody binding to Fcγ receptors is described in several publications. A role for FcγRIIIA in clinical efficacy of trastuzumab was published in a study on FcγR polymorphisms in trastuzumab‐treated patients with HER2‐positive metastatic breast cancer 45. The efficacy differences found are attributed to FcγRIIIa V158/F158 polymorphism, for which a 2‐ to 2.5‐fold difference in affinity for monoclonal human IgG1 is reported 46. A similar effect has been reported for fucosylated and nonfucosylated rituximab with respect to FcγRIIIa and FcγRIIIb binding, where a twofold difference in binding affinity was measured 47. It is attractive to speculate that the differences that we found may also potentially have clinical relevance as they span twofold differences.

High‐throughput analytical screening technologies are used more and more to rapidly identify critical process parameters and to monitor critical product quality attributes. Here, we have shown that Fc binding assays can be applied for a rapid screening of product quality. Understanding the effects of process variation on Fc tail functionality early in the development can be beneficial for further process development, in lead optimization studies, and in process characterization studies. Although ideally the Fc receptors screening should be performed on a single SPR assay, the differences in binding characteristics between the various receptors prevented such a multiplexed measurement. However, three separate high‐throughput screening methods were developed and used to explore the total Fc region binding of stressed IgGs. Low‐affinity Fcγ receptors and FcRn binding were measured in only 5 min per sample, whereas the FcγRI assay takes 45 min per sample; especially, the screening of multiple Fcγ receptors in a single assay with only 5 min per sample dramatically increases sample throughput, and therefore, such multiplexed methods are highly recommended to use.

Although the various stress‐induced modifications are considered to be crucial for product quality, we here show that surprisingly most of those factors had only minor effects on FcRn binding within the range that is often found during development. This is relevant for the development of novel antibodies but has even more impact on the development of biosimilar antibodies. During the development of biosimilars, due to process difference with the innovator, small differences occur in, for example, level of Ox or deamidation, for which the question always remains whether they are relevant for product quality. Biosimilarity assessment can be rapidly made using such high‐throughput screening assays. Here, we show that only significant differences in these parameters impacted FcRn binding and minute changes had no impact at all, except for minor differences in the presence of HMW species. Furthermore, as the future of biotherapeutic developments is evolving to continuous manufacturing strategies, such screening technologies as presented here are in improvement to rapidly monitor product quality in near real time.

## Author contributions

ME designed the project. KG and CO developed the assays and acquired the data. KG, CO, and DE analyzed and interpreted the data. KG wrote the manuscript, and DE, PS, LB, RS, and ME reviewed the manuscript.

## Supporting information


**Fig. S1**. Overlaid 280 nm chromatograms of purified monomeric deamidated sample, directly after preparative SEC (black) and after one freeze‐thaw cycle overnight (blue).
**Fig. S2**. Relative binding on the four low affinity Fcγ receptors with the deamidated sample before and after SEC purification.
**Fig. S3**. Boxplots of apparent affinity of stressed samples immobilized on the sensor surface and Fcγ receptors injected as analytes.
**Fig. S4**. Preparative SEC chromatogram at 280 nm of collected fractions (A) and corresponding SDS‐PAGE analysis of the collected fractions (B).
**Fig. S5**. Single cycle kinetics sensorgrams of purified monomer (A), dimer (B) and oligomer (C) fractions on FcγRI binding.
**Fig. S6**. Sensorgrams of a monomeric IgG1 sample (40 nM) in overlay with covalent dimer and multimer samples on FcRn binding.
**Fig. S7**. Three‐dimensional model of an IgG1 with the residues that are involved in Fc interactions indicated in yellow, pink and blue.
