# Supplementary material for: Rapid screening of IgG quality attributes – effects on Fc receptor binding
Source: FEBS Open Bio. 2017 Sep 5;7(10):1557–74. doi: 10.1002/2211-5463.12283 (PMC5623700; doi:10.1002/2211-5463.12283)
Supplement: Supplementary file 1 — Fig. S1. Overlaid 280 nm chromatograms of purified monomeric deamidated sample, directly after preparative SEC (black) and after one freeze‐thaw cycle overnight (blue). Fig. S2. Relative binding on the four low affinity Fcγ receptors with the deamidated sample before and after SEC purification. Fig. S3. Boxplots of apparent affinity of stressed samples immobilized on the sensor surface and Fcγ receptors injected as analytes. Fig. S4. Preparative SEC chromatogram at 280 nm of collected fractions (A) and corresponding SDS‐PAGE analysis of the collected fractions (B). Fig. S5. Single cycle kinetics sensorgrams of purified monomer (A), dimer (B) and oligomer (C) fractions on FcγRI binding. Fig. S6. Sensorgrams of a monomeric IgG1 sample (40 nM) in overlay with covalent dimer and multimer samples on FcRn binding. Fig. S7. Three‐dimensional model of an IgG1 with the residues that are involved in Fc interactions indicated in yellow, pink and blue. [file FEB4-7-1557-s001.docx]

**Rapid screening of IgG quality attributes: effects on Fc receptor binding**

Karin P.M. Geuijen* ^a,b^, Cindy Oppers-Tiemissen^c^, David F. Egging^c^, Peter J. Simons^d^, Louis Boon^d^, Richard B.M. Schasfoort^e^, Michel H.M. Eppink^a,b^

^a^ Downstream processing, Synthon Biopharmaceuticals BV, PO Box 7071, 6503 GN Nijmegen, the Netherlands

^b^ Bioprocess Engineering, Wageningen University, PO Box 16, 6700 AA Wageningen, the Netherlands

^c^ Preclinical department, Synthon Biopharmaceuticals BV, PO Box 7071, 6503 GN Nijmegen, the Netherlands

^d^ Bioceros BV, Yalelaan 46, 3584 CM, Utrecht, the Netherlands

^e^ Medical Cell Biophysics group, MIRA institute, Faculty of Science and Technology, University of Twente, PO Box 217, 7500 AE Enschede, the Netherlands

**Supporting information**

Supporting Figure 1 Overlaid 280 nm chromatograms of purified monomeric deamidated sample, directly after preparative SEC (black) and after one freeze-thaw cycle overnight (blue). The small peak in front of the main peak corresponds to HMW species.

Supporting Figure 2 Relative binding on the four low affinity Fcγ receptors with the deamidated sample before and after SEC purification

Supporting Figure 3 Boxplots of apparent affinity of stressed samples immobilized on the sensor surface and Fcγ receptors injected as analytes. Steady state equilibrium affinity (SSE KD) determined for (A) FcγRIIIa, (B), FcγRIIIb, (C) FcγRIIa, (D) FcγRIIb. Kinetic 1:1 fit to determine affinity for (E) FcγRI. Note that the Y-axis for graph E, FcγRI, is on logarithmic scale.

Supporting Figure 4 Preparative SEC chromatogram at 280 nm of collected fractions (A) and corresponding SDS-PAGE analysis of the collected fractions (B). The fractions from B1 to C4, indicated in black in the chromatogram, were collected and analyzed.

Supporting Figure 5 Single cycle kinetics sensorgrams of purified monomer (A), dimer (B) and oligomer (C) fractions on FcγRI binding. Measured curves in green or red, fitted curves in black. Dimer and oligomer samples have a slower off-rate compared to the monomer as a result of altered kinetics of the interaction.

Supporting Figure 6 Sensorgrams of a monomeric IgG1 sample (40 nM) in overlay with covalent dimer and multimer samples on FcRn binding. Dimer and oligomer samples have a slower off-rate compared to the monomer as a result of altered kinetics of the interaction.

Supporting Figure 7 Three-dimensional model of an IgG1 with the residues that are involved in Fc interactions indicated in yellow, pink and blue. The asparagine in the Fc region of the IgG that is most prone towards deamidation is indicated in red.
